# GABA Maintains the Proliferation of Progenitors in the Developing Chick Ciliary Marginal Zone and Non-Pigmented Ciliary Epithelium

**DOI:** 10.1371/journal.pone.0036874

**Published:** 2012-05-09

**Authors:** Henrik Ring, Suresh Kumar Mendu, Shahrzad Shirazi-Fard, Bryndis Birnir, Finn Hallböök

**Affiliations:** Department of Neuroscience, Uppsala University, Uppsala, Sweden; Indiana University School of Medicine, United States of America

## Abstract

GABA is more than the main inhibitory neurotransmitter found in the adult CNS. Several studies have shown that GABA regulates the proliferation of progenitor and stem cells. This work examined the effects of the GABA_A_ receptor system on the proliferation of retinal progenitors and non-pigmented ciliary epithelial (NPE) cells. qRT-PCR and whole-cell patch-clamp electrophysiology were used to characterize the GABA_A_ receptor system. To quantify the effects on proliferation by GABA_A_ receptor agonists and antagonists, incorporation of thymidine analogues was used. The results showed that the NPE cells express functional extrasynaptic GABA_A_ receptors with tonic properties and that low concentration of GABA is required for a baseline level of proliferation. Antagonists of the GABA_A_ receptors decreased the proliferation of dissociated E12 NPE cells. Bicuculline also had effects on progenitor cell proliferation in intact E8 and E12 developing retina. The NPE cells had low levels of the Cl–transporter KCC2 compared to the mature retina, suggesting a depolarising role for the GABA_A_ receptors. Treatment with KCl, which is known to depolarise membranes, prevented some of the decreased proliferation caused by inhibition of the GABA_A_ receptors. This supported the depolarising role for the GABA_A_ receptors. Inhibition of L-type voltage-gated Ca^2+^ channels (VGCCs) reduced the proliferation in the same way as inhibition of the GABA_A_ receptors. Inhibition of the channels increased the expression of the cyclin-dependent kinase inhibitor p27^KIP1^, along with the reduced proliferation. These results are consistent with that when the membrane potential indirectly regulates cell proliferation with hyperpolarisation of the membrane potential resulting in decreased cell division. The increased expression of p27^KIP1^ after inhibition of either the GABA_A_ receptors or the L-type VGCCs suggests a link between the GABA_A_ receptors, membrane potential, and intracellular Ca^2+^ in regulating the cell cycle.

## Introduction

In many vertebrates, the development of the retina is not complete after the embryonic period. New neurons can be generated from stem cells or stem cell-like cells during postnatal development or, for some species, throughout life [1]. Neurons can also be generated from cells with stem cell properties after injuries. Three stem cell- or stem cell-like sources have been identified in the eye: Müller cells, cells of the ciliary body and cells of the ciliary marginal zone (CMZ) [2]–[6]. A fourth source of new neurons is the retinal pigment epithelium [7], [8]. However, neurogenesis from the pigment epithelium requires dedifferentiation [9]. This study focuses on the regulation of the proliferation of retinal progenitor cells and of cells from the non-pigmented epithelium of the chicken ciliary body. Chicken non-pigmented ciliary epithelial (NPE) cells are derived from the optic cup neuroepithelium and share similarities with early retinal progenitors such as the expression of Pax6 and Chx10 [4]. The cells can be stimulated by exogenous growth factors to proliferate and generate neurons *in vivo* [4], and if NPE cells are dissociated and cultured they form neurospheres that express many retinal progenitor cell markers [10]. The stem cell properties of ciliary epithelial cells have been challenged [10], [11]. Neurons generated from the chicken NPE do not integrate into the neural retina [4] and are probably not an endogenous source for retinal regeneration. However, they have the potential to be harvested, cultured and then transplanted back into injured retinas to replace lost neurons [12].

Neural stem cells persist in specialised niches with a cellular milieu that supports their pluripotency and regulates their proliferation. Many well-known factors engage in regulating cell proliferation; cadherin-mediated contact growth inhibition, integrin-mediated attachment to the basal lamina, and responsiveness to extrinsic signals such as growth factors, Wnts and sonic hedgehog [13]–[15]. However our knowledge of what regulates stem cell proliferation in these niches is still rather limited. There is evidence that the plasma cell membrane potential influences cell proliferation [16], [17] and factors, such as neurotransmitters, that change the membrane potential contribute to the control of cell proliferation [18]. One of the classical neurotransmitters, γ-aminobutyric acid (GABA), has been shown to regulate proliferation of several cell types including embryonic stem cells [19], cortical progenitor cells [20], [21] and immune cells [22], [23]. GABA_A_ receptors are GABA-gated Cl^−^ channels that mediate fast synaptic inhibitory neurotransmission in the mature mammalian CNS [24]. These receptors are heteropentameric assemblies that often contain 2α, 2β and 1γ or δ subunits [24], [25]. In chicken 19 different subunits have been identified: 6 alpha (α1–6), 4 beta (β1–4), 3 gamma (γ1–3), delta (δ), epsilon (ε), pi (π) and 3 rho subunits (ρ1–3) [26]. Neurons express different sets of subunits giving rise to channels with different functional and pharmacological properties [27]. GABA_A_ receptors are not only present on neurons in inhibitory synapses but are also found outside synapses and on non-neural cells. Such extrasynaptic receptors have high affinity for GABA and open the Cl^−^ channels during sustained periods at low ambient GABA concentrations (1 µM). This leads to changes in the membrane potential (tonic inhibition) [28]. Many embryonic cells including neuronal progenitors have high intracellular Cl^−^ concentration. Opening the GABA_A_ receptor Cl^−^ channels will therefore lead to Cl^−^ efflux and depolarisation of the membrane [29].

This study shows that chicken NPE cells express extrasynaptic-like GABA_A_ receptors that are involved in regulating the proliferation of the cells. Inhibition of GABA_A_ receptors decreased the proliferation of dissociated NPE cells and of retinal progenitors in the intact E8 retina but not of progenitors in E3.5 or E5 retina. The results suggest that GABA_A_ receptor driven changes in the membrane potential activate L-type voltage gated Ca^2+^ channels (VGCC), and that inhibition of the channels causes an increased expression of the cyclin-dependent kinase inhibitor (CDI) p27^KIP1^.

## Materials and Methods

### Ethics statement

This study was carried out in strict accordance with the recommendations in the Guide for the Care and Use of Laboratory Animals of the Association for Research in Vision and Ophthalmology. The protocol was approved by the Committee on the Ethics of Animal Experiments by Uppsala djurförsöksetiska nämnd (permit number C11/9).

### Animals

Fertilised White Leghorn eggs were obtained from Ova Produktion AB (Västerås, Sweden) and incubated at 38°C in a humidified incubator. Embryos were staged (st) according to Hamburger and Hamilton [30].

### Dissection and culturing of NPE cells

Embryonic day (E) 12 (st38) chicken eyes were used because the E12 NPE is easily dissected as a pure structure without pigmented cells, and that E12 is after the period of massive proliferation in the retina. A standard cell preparation started out with material from 20 embryos. Eyes were placed with the lens facing down. The eye was cut equatorially at one quarter of the distance between the lens and the fovea. The retinal pigment epithelium and retina were peeled back exposing the vitreous body with the NPE attached to the vitreous. The NPE was stripped loose using forceps and it was collected with a pipette. The cells were dissociated by repeated trituration in cell culture medium with a glass pipette held tightly against the bottom of the tube. The cells were cultured in DMEM-F12 (Invitrogen, Grand Island, NY, USA; cat. no. 31331028) with 2% B-27 Serum-Free Supplement (50×) (Invitrogen, cat. no. 17504-044) and at 37°C in 5% CO_2_. For whole eye explant cultures, E3.5 (st22), E5 (st27), E8 (st35) and E12 chicken eyes were stripped from the sclera and then cultured in DMEM-F12 with 5% FCS and incubated at 37°C in 5% CO_2_.

### Quantitative reverse transcription PCR

Total RNA was isolated from E12 NPE cells by using TRIzol reagent (Invitrogen, cat. no. 15596-018). Four RNA preparations from NPE cells were collected. Complementary DNA (cDNA) was prepared from 1 µg of RNA using GeneAmp (Applied Biosystems, Carlsbad, CA, USA).

The quantitative reverse transcription PCR (qRT-PCR) analysis was performed using IQ™ SyBr Green Supermix (Biorad, Herculus, CA, USA; cat. no. 170-8884) with primers designed by using Primer Express v2.0, default setting; Tm 60°C, 50% G/C, and amplicon size minimum 100 base pairs. Each primer sequence was blasted separately against GenBank and EMBL and only primers with a perfect match within the target sequence and with the second best hit <75% identity, were used. To confirm identity of amplified PCR products, dissociation curve analyses and agarose gel electrophoresis were performed. Primers used: p21^CIP^ (NM_204396) 5′-caatgccgagtctgtagttccc-3′ and 5′-ttccagtcctcctcagtccctt-3′, p27^KIP1^ (ENSGALT00000023032, NM_204256) 5′-ccgtcagagcctggatgtaaa-3′ and 5′-catcagtctttcggcctacaca-3′, GAD65 (ENSGALT00000012268, XM_418596) 5′- atggtgagctatcagcctctgg -3′ and 5′- ccaggcgctctatttcatcaa -3′, GAD67 (ENSGALT00000015628, NM_204913) 5′-gtcgaataaagatggcgatgga-3′ and 5′- cagccatgcctttggttttg-3′; for primer sequences of GABA_A_ receptor subunits, NKCC1 and KCC2 see previous work [26]. The initial mRNA levels were normalised to β-actin and TATA-box binding protein (TBP) mRNA levels in order to correct variations in the cDNA syntheses [26]. The statistical analysis used for the qRT-PCR data was analysis of variation (ANOVA); Tukey's multiple comparison post-hoc test.

### Electrophysiology

Whole-cell patch-clamp recordings were performed to monitor the effects of GABA on freshly dissected NPE cells. The cells were washed with extracellular recording solution containing in mM: 145 NaCl, 5 KCl, 1 MgCl_2_, 1.8 CaCl_2_ and 10 TES pH 7.4. GABA and SR-95531 were dissolved in the extracellular recording solution. The cells were either treated with 1 µM or 100 µM of GABA. The pipette solution contained in mM: 125 KCl, 5 CsCl, 1 MgCl_2_, 1.8 CaCl_2_, 5 EGTA and 10 TES pH 7.4. The pipette holding potential was −90 mV. Pipettes were made of borosilicate glass and the pipette resistance used for the whole-cell recordings ranged from 4 to 8 mÙ. The average current was measured as the average of deviation of all data points from the middle of the baseline current. The whole-cell currents were recorded with an Axopatch 200B amplifier (Molecular devices, Palo Alto, CA, USA), filtered at 2 kHz, digitised at 10 kHz using a digiData 1322A analogue-to-digital converter interfaced with a computer and analysed by pClamp 9.2 software.

### Proliferation analyses: [ ^3^H]-thymidine incorporation, EdU and MTT assays

Each *in vitro* analysis contained pooled NPEs from 10–20 animals. Dissociated NPE cells were incubated for 16 hours in media without reagents. The cells were then treated with 1 µM GABA (Tocris, Bristol, UK; cat. no. 0344), 50 µM muscimol (Tocris, cat. no. 0289), 20 µM bicuculline methiodide (Sigma-Aldrich, St. Louis, MO, USA; cat. no. 14343), 50 µM SR-95531 (Sigma-Aldrich, cat. no. S106), 50 µM picrotoxin (Tocris, cat. no. 1128) or 10 µM nifedipine (Tocris, cat. no 1075) (Table 1). The reagents were added with [^3^H]-thymidine (final concentration 0.005 µCi/µl; Perkin-Elmer, Waltham, MA, USA; cat. no. Net027X001MC) to measure DNA synthesis during 16 hours. All reagents were dissolved according to the manufacturer's recommendations. [^3^H]-thymidine incorporation was examined (4 biological replicates per treatment) by harvesting the cells to a glass fibre filtermat (Perkin-Elmer, cat. no. 1205-401). This was analysed in a Wallac 1205 Betaplate Liquid Scintillation counter (Wallac, Turku, Finland). Some of the results were verified by using the thymidine analogue 5-ethynyl-2′-deoxyuridine (EdU; final concentration 0.2 mM; included in Click-iT EdU Alexa Fluor 488 imaging kit, Invitrogen, cat. no. C10337) or a 3-(4,5-dimethylthiazol-2-yl)-2,5-diphenyltetrazolium bromide (MTT; final concentration 0.5 µg/µl; Sigma-Aldrich, cat. no. M-2128) assay. The MTT assay was performed according to the manufacturer's protocol and analysed on a Multiskan MS plate reader (Labsystems, Vantaa, Finland). The EdU was detected according to the manufacturer's protocol and EdU positive and negative cells were manually counted by using a Zeiss Axioplan2 microscope equipped with Axiovision software (Carl Zeiss Vision GmbH, Göttingen, Germany; software version 4.8). 1.5 µg/ml FGF-2 (Peprotech, London, UK; cat. no. 100-18B) was used as a positive control. The statistical analysis used in the proliferation assays was one-way ANOVA; Tukey's multiple comparison post-hoc test.

**Table 1:** The stimulators and inhibitors used in the study and their modes of action.

Target	Chemical	Reference	Action
L-type VGCC	nifedipine	[68], [69]	L-type calcium channel blocker
GABA_A_ receptor	muscimol	[70]	selective GABA_A_ receptor agonist
GABA_A_ receptor	bicuculline methiodide	[71]	competitive GABA_A_ receptor antagonist
GABA_A_ receptor	picrotoxin	[72]	non-competitive GABA_A_ receptor antagonist
GABA_A_ receptor	SR-95531	[73]	specific GABA_A_ receptor antagonist
ATM/ATR	CGK733	[74]	kinase inhibitor
Chk1	SB 218078	[75]	kinase inhibitor. ATP-competitive inhibitor of Chk1

Trypan blue exclusion test of cell viability was used to analyse cell death/survival in treated (1 µM GABA and 50 µM bicuculline) and untreated (control; 1 µM GABA) NPE cells. 0.4% trypan blue cell suspensions (1∶1) were mixed (4 biological replicates) and incubated for 3 minutes at room temperature. Unstained (viable) and stained (nonviable) cells were counted. Cell death/survival was also analysed by flow cytometry and immunocytochemistry.

### Flow cytometry

Fluorescence-activated cell sorting (FACS) was used to study the distribution of cells in the different phases of the cell cycle as well as apoptosis of the NPE cells. Dissociated NPE cells were treated with either 50 µM bicuculline with 1 µM GABA or 1 µM GABA (control) over night and fixed in 80% methanol. The cells were either directly stained with 50 µg/ml propidium iodide (PI, Sigma-Aldrich, cat. no. P-4170), 0.1% Triton ×100 and 20 µg/ml RNase A in phosphate-buffered saline (PBS) or treated with DNA extraction buffer (0.2 M Na_2_HPO_4_ and 0.1 M citric acid) and then stained with PI. The samples were run on a BD LSR II flow cytometer (BD Biosciences, Franklin Lakes, NJ USA) using FACSDiva version 6.0 software and analysed by the ModFit LT DNA analysis software (Verity Software house, Topsham, ME, USA; version 3.2.1; Model 1Dn0n-DSD). The ModFit DNA analysis was based on 12 000 events/treatment (4 biological replicates/treatment). Fragmented DNA in apoptotic cells with less DNA than G_1_ cells was detected by FACS analysis.

### Immunocytochemistry

Cells were plated on poly-ornithine-hydrobromide- (Sigma-Aldrich; cat. no. P3655) and fibronectin- (Invitrogen; cat. no. 33010018) coated 13 mm cover glasses for 2 hours and then fixed in 4% paraformaldehyde in PBS for 10 minutes at 37°C. Cells were incubated in blocking solution (PBS containing 1% fetal calf serum and 0.1% Triton X-100) for 10 minutes. Primary and secondary antibodies were diluted in blocking solution. Primary antibodies were allowed to react with the fixed cells over night at 4°C, and secondary antibodies for 2 hours at room temperature. Primary antibodies used in this study were against γ-H2AX (Abcam, Cambridge, UK; cat. no. ab11174; 1∶4 000), GABA (Sigma-Aldrich cat. no. A0310; 1∶1 000), p27^KIP1^ (BD Transduction Labs, Stockholm, Sweden; cat. no. 610241; 1∶200) and cleaved caspase-3 (Cell signaling, Danvers, MA, USA; cat. no. 9661; 1∶4 000). Alexa Flour conjugated secondary antibodies were obtained from Invitrogen.

The cells were analysed by using a Zeiss Axioplan2 microscope equipped with Axiovision software. All plated cells were counted and analysed. As a positive control for the γ-H2AX antibody NPE cells were treated with neocarzinostatin (0.2 µg/ml; Sigma-Aldrich, cat. no. N9162) for 30 minutes. The statistical test was one-way ANOVA; Tukey's multiple comparison post-hoc test.

### Immunohistochemistry

Eyes from E12 and retinal explants, from E3.5, E5, E8 and E12, were fixed in 4% paraformaldehyde in PBS for 15 minutes at 4°C, incubated for 3 hours in 30% phosphate-buffered sucrose at 4°C, embedded in OCT freezing medium (Sakura, Alphen aan den Rijn, The Netherlands), frozen and sectioned in a cryostat. Retinas were cryosectioned sagittally through the lens producing 10 µm dorsal to ventral sections of the retina. Primary antibodies were incubated over night at 4°C, and secondary antibody for 2 hours at room temperature. Primary antibodies were against GABA, Ap2α (Hybridoma bank, Iowa city, Iowa, USA), Pax6 (Hybridoma bank) and Isl1 (Hybridoma bank). The EdU incorporation was detected according to the manufacturer's protocol and EdU positive and negative cells were manually counted. Images were captured with a Zeiss Axioplan2 microscope equipped with Axiovision software. The statistical tests were Student's t and Mann-Whitney.

## Results

### GABA_A_ receptors on NPE cells

**Figure 1:**
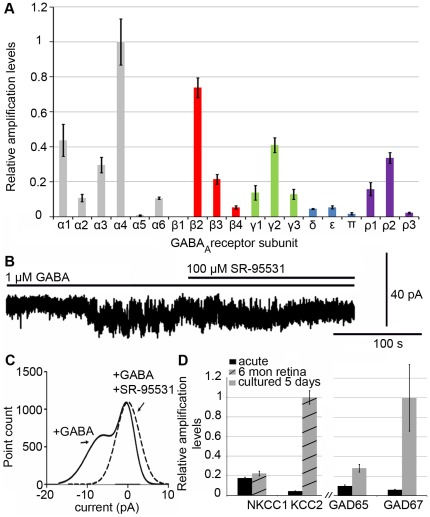
Characterisation of the GABA_A_ receptor system in NPE cells. (A) Relative qRT PCR amplification levels of the 19 GABA_A_ receptor subunit mRNA in NPE cells. Grey columns for α subunits, red columns for β subunits, green columns for γ subunits, blue columns for δ, ε, π subunits and purple columns for ρ subunits. Error bars ±SD, n = 4 independent preparations each containing a pool of more than 10 NPE. (B) Electrophysiology of dissociated NPE cells. 1 µM GABA activated currents (−90 mV holding potential) that were inhibited by application of the GABA_A_ receptor antagonist SR-95531 (100 µM). n = 5. (C) Gaussian fits to all-points histograms derived from the current record shown in (B): solid line, currents after GABA application; broken line, currents after application of SR-95531. The difference between the two peaks in the presence of GABA equals the mean tonic current (−6.2 pA). (D) Relative qRT PCR amplification levels of NKCC1, KCC2, GAD65 and GAD67 mRNA in acute NPE cells compared to 6 months old retina (NKCC1 and KCC2) or cultured NPE cells (GAD65 and GAD67). Error bars ±SD, n = 4 as above.

The expression of GABA_A_ receptor subunits in NPE cells were studied by using qRT-PCR. 17 subunits were expressed above background levels with the α1, α3, α4, β2, γ2 and ρ2 subunits showing the highest mRNA levels (Fig. 1A). α5 and β1 subunits were not expressed (Fig. 1A).

To examine if the GABA_A_ receptors were functional, dissociated NPE cells were analysed using the patch-clamp technique. GABA was applied and the activation of GABA_A_ receptor Cl^−^ channels was recorded. 1 µM GABA activated currents in the cells that could be inhibited by the GABA_A_ receptor competitive antagonist SR-95531 (n = 5; Fig. 1B). GABA activated the currents after a delay, which is consistent with an extrasynaptic-like nature of the receptors [31]. Figure 1C shows Gaussian fits to histograms generated from the current record shown in Figure 1B. The first peak represents the baseline current and the second peak is the most frequent GABA-activated current. The difference between the two peaks, in the presence of GABA, is the mean GABA-activated current (−6.2 pA). Similar currents were obtained in 5 cells giving the average GABA-activated current of −4.5±1.39 pA (n = 5, hp = −90 mV).

### Expression of NKCC1 and KCC2 in NPE cells

Increased expression of the chloride co-transporter KCC2 during CNS development is a key event in the shift from high to low intracellular Cl^−^ concentrations [32] and, therefore, for the shift from excitatory (depolarising) to inhibitory (hyperpolarising) actions by the GABA_A_ receptor signalling system [33]. The relative expression of NKCC1 and KCC2 mRNA in NPE cells was analysed. Both co-transporters were expressed at low levels in the NPE cells. The relative amplification levels of NKCC1 were approximately 4-fold higher than those of KCC2 (Fig. 1D). The relation suggests that these cells have a net Cl^−^ influx resulting in a relative high intracellular Cl^−^ concentration. In the mature retina, KCC2 mRNA expression is much higher compared to that of NKCC1 (Fig. 1D) [26].

### NPE cells express low levels of GAD65, GAD67 and GABA

The subunit expression and the GABA-activated currents showed that the NPE cells have functional GABA_A_ receptors. The next question was if the GABA_A_ receptors could modulate NPE cell proliferation. Dissociated E12 NPE cells were grown in the presence of [^3^H]-thymidine to examine effects on cell proliferation. Cells were cultured over night before [^3^H]-thymidine was added to the cultures and after 16 hours of incubation the cells were examined for incorporated [^3^H]-thymidine into the DNA.

The [^3^H]-thymidine incorporation varied substantially between different cell preparations and cultures (data not shown). The variation was abolished and the proliferation stabilised in presence of 1 µM GABA. This effect could be attributed to endogenous, variable GABA synthesis in the cultures. We confirmed this hypothesis by analysing the expression of the GABA synthesising enzymes GAD65 and GAD67 [34]. We found low but increased mRNA levels in cultured NPE cells. The expression increased with time in culture (Fig. 1D). The number of GABA positive cells in freshly dissected NPE cells was less than 2% (15 of 789 cells) but this number increased to over 30% (298 of 925 cells) after 5 days in culture (data not shown). These results showed that a subset of the dissociated NPE cells started to produce GABA with increasing time in culture, which may reflect cell differentiation. All subsequent analyses were therefore performed in the presence of 1 µM GABA during the 16 hours of incubation. These results showed that the freshly dissociated NPE cells proliferate in the presence of GABA.

### GABA_A_ receptor antagonists decrease cell proliferation

**Figure 2:**
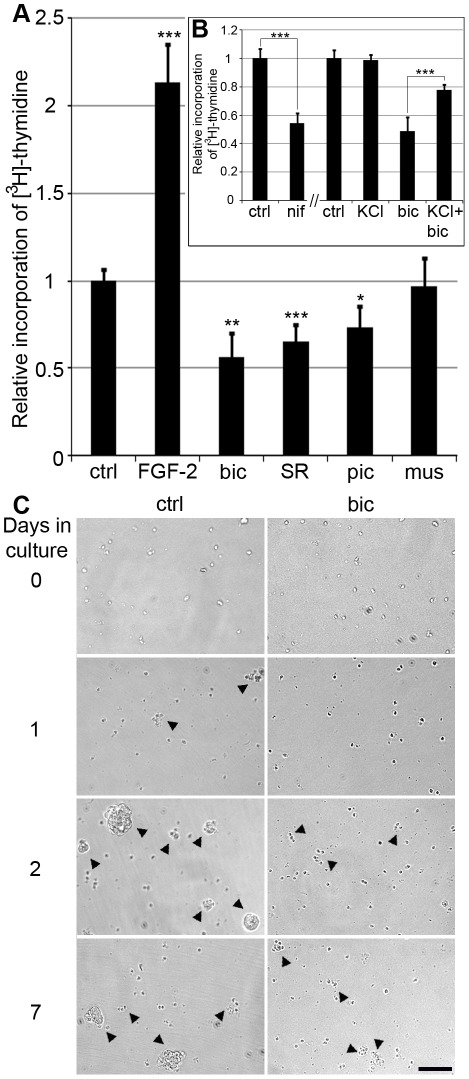
Effects of GABA_A_ receptor and voltage-gated Ca^2+^ channel inhibitors on NPE cell proliferation. Bar graphs show the relative proliferation levels of dissociated NPE cells determined by incorporation of [^3^H]-thymidine. (A) Proliferation levels of cells treated with FGF-2 (1.5 µg/ml), bicuculline (20 µM bicuculline, 1 µM GABA), SR-95331 (50 µM SR-95531, 1 µM GABA), picrotoxin (50 µM picrotoxin, 1 µM GABA) and muscimol (50 µM muscimol, 1 µM GABA) in relation to control cells (1 µM GABA), (B) Proliferation levels of cells treated with the VGCC antagonist nifedipine (10 µM nifedipine, 1 µM GABA), KCl (20 mM, 1 µM GABA), bicuculline (20 µM, 1 µM GABA) or KCl + bicuculline (20 µM bicuculline, 20 mM KCl, 1 µM GABA) in relation to control cells (1 µM GABA). Vehicle and control for nifedipine treatment was DMSO (0.01%). Error bars ±SD, n = 4 independent cultures. Statistical test was one-way ANOVA, Tukey's multi-comparison post-hoc test; p<0.05*, p<0.01**, p<0.001***. (C) Bright-field phase contrast micrographs of cultured dissociated control and bicuculline-treated NPE cells. Arrowheads point at initial clusters and spheres with proliferating cells. bic, bicuculline; ctrl, control; FGF-2, basic fibroblast growth factor; mus, muscimol; nif, nifedipine; pic, picrotoxin; SR, SR-95531. Scale bar in (C) is 100 µm.

Dissociated NPE cells were treated with the GABA_A_ receptor agonist muscimol, and the antagonists bicuculline, SR-95531 and picrotoxin. FGF-2 was used as a positive control. The proliferation was analysed by [^3^H]-thymidine incorporation. The effects were also analysed by MTT assay and by cytochemical analysis of EdU incorporation. The positive control FGF-2, known to increase the proliferation of NPE cells [4] increased [^3^H]-thymidine incorporation 2-fold (Fig. 2A).

The GABA_A_ receptor agonist muscimol did not further increase the proliferation when added to 1 µM GABA (Fig. 2A). In contrast, the GABA_A_ receptor antagonist bicuculline decreased the proliferation 1.8-fold compared to control (1 µM GABA) (Fig. 2A). The decrease was confirmed by using EdU and MTT assays. Untreated NPE cells formed non-adherent spheres in culture and treatment with bicuculline inhibited the formation of spheres compared to control cells (Fig. 2C). The GABA_A_ receptor antagonist SR-95531 decreased the proliferation 1.5-fold compared to control (Fig. 2A), which also was confirmed by EdU and MTT assays (data not shown). A third GABA_A_ receptor antagonist, picrotoxin, decreased the proliferation 1.4-fold compared to control (Fig. 2A).

In order to study if the bicuculline treatment had irreversible effects on the cell proliferation, bicuculline was washed out and treated cells were analysed to see if they could reinitiate their proliferation. Cytological examination of EdU-incorporation in the presence of 1 µM GABA showed that 23±5% (1031 of 4520 cells; n = 4) of the cells were EdU positive and had gone through S-phase during the analysis period for 16 hours. NPE cells were treated with bicuculline (16 hours) and one half of the cultures were washed with PBS and cultured in new medium with 1 µM GABA added. The remaining cultures had bicuculline. The proliferation was assessed by EdU incorporation. 18±2% (n = 4) of the washed cells and 6±3% (n = 4) of the bicuculline-treated cells were EdU positive showing that bicuculline-treated cells retained their proliferative capacity if the bicuculline inhibition is lifted.

To further exclude the possibility that the decreased proliferation was a result of cell death, a trypan blue exclusion test of cell viability was performed. The number of cells excluding trypan blue was similar in cells treated with bicuculline as in control (1 µM GABA). The cells were also stained with PI and analysed by FACS. Apoptosis can be detected on a content frequency histogram as a “sub-G_1_ peak” but the results showed no differences of sub-G_1_ peaks between bicuculline-treated and control cells (data not shown). Immunocytochemistry for caspase-3 did not reveal differences between bicuculline-treated and control cells (data not shown). We concluded that the decreased proliferation was not due to decreased cell viability or cell death.

### Bicuculline decreases cell proliferation in the intact retina

**Figure 3:**
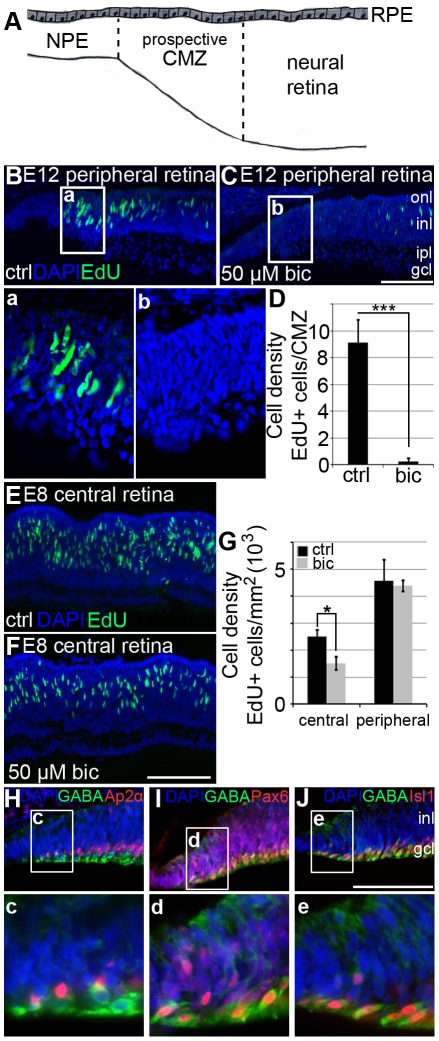
GABA_A_ receptor mediated effects on retinal progenitor cell proliferation. (A) Schematic diagram of the E12 peripheral retina. Cells between the dashed lines in the prospective ciliary marginal zone (CMZ) on the dorsal side of the retina were counted. Cells that had gone through S-phase were detected by EdU labelling. Fluorescence micrographs of EdU labelled cells in (B) untreated (control) and (C) bicuculline-treated E12 retinal explants cultured for 4 hours. Images in (a) and (b) show the boxed regions in (B) and (C). (D) Bar graph shows the cell density of EdU positive cells per CMZ in control and bicuculline-treated explants. Error bars ± S.D. n = 4 explant cultures, average of 4 sections per explant, two-tailed Student t-test; *** p<0.001. Fluorescence micrographs of EdU labelled cells in the central retina in (E) control and (F) bicuculline-treated E8 explants cultured for 4 hours. (G) Bar graph shows cell density of EdU positive cells in central and peripheral retina in control (black bars) and bicuculline-treated (grey bars) E8 explants. Error bars ± S.D. n = 3 explants cultures, average of 4 sections per explant, Mann-Whitney test; * p = 0.05. (H–J) GABA+ cells close and within the prospective CMZ labelled for (H) Ap2α, (I) Pax6 or (J) Isl1. Images in (c) to (e) show the boxed regions in (H–J) in higher magnification. gcl, ganglion cell layer; inl, inner nuclear layer; ipl, inner plexiform layer; onl, outer nuclear layer; RPE, retinal pigment epithelium. Scale bar in (C) is 100 µm and is valid for (B). Scale bar in (F) is 100 µm and is valid for (E). Scale bar in (J) is 100 µm is valid for (H–J).

To examine if GABA_A_ receptors modulate proliferation within the intact retina, eye explants were treated with 50 µM bicuculline and the proliferation was studied by immunohistochemistry after 4 hours of EdU incorporation. Cells situated in the NPE (Fig. 3A) do not divide or divide very seldom *in situ*. Therefore, we analysed the neighbouring cells in the prospective CMZ of the same age (E12). Cells in the same prospective CMZ region, the dorsal portion of the far peripheral retina, were counted in the samples to avoid differences introduced by the uneven spatiotemporal developmental of the CMZ [35]. The proliferation of cells in the prospective CMZ was lower in bicuculline-treated eyes (0.25±0.29 EdU positive cells/CMZ; n = 4), compared to control eyes (9.11±1.7 EdU positive cells/CMZ; n = 4) (Fig. 3B–D).

Retinal progenitors from E3.5, E5 and E8 retinas were also studied. Eye explants were treated and analysed in a similar way as the E12 explants. The central but not peripheral retina in the E8 explants was affected by bicuculline (1247±139 cells/mm^2^; n = 3) compared to control retinas (1972±372 cells/mm^2^; n = 3) (Fig. 3E–G). However, there were no significant effects on EdU incorporation in E3.5 or E5 bicuculline-treated explants compared to control (supplementary figure S1).

### GABAergic cells within the prospective E12 CMZ are Pax6+ and Isl1+

NPE cells have a low endogenous expression of GABA synthesizing enzymes (Fig. 1D). GABA-ergic cells in the vicinity on the prospective CMZ were identified by immunohistochemistry for GABA, Pax6, Ap2α, or Isl1. GABA, Ap2α double positive cells were seen in the retina but not in the periphery or within the prospective CMZ (Fig. 3H). GABA+ cells within the prospective CMZ were Pax6+ or Isl1+ and were situated on the vitreal side in the prospective ganglion cell layer (Fig. 3I and J).

### Cell cycle analysis of NPE cells after treatment with bicuculline

**Figure 4:**
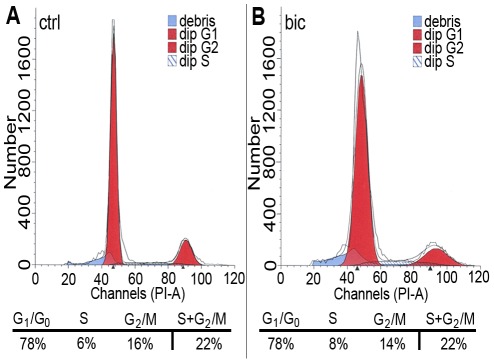
Distribution of NPE cells in the cell cycle phases after treatment with bicuculline. Flow cytometry analysis of PI labelled NPE cells. Graphs are showing PI fluorescence intensity (DNA content) per event. (A) Control cells (1 µM GABA) and (B) bicuculline-treated cells (20 µM bicuculline, 1 µM GABA). Red peaks from left: G_1_-, and G_2_-phase. Tables list the percentage of cells present in the cell cycle phases calculated by the ModFit LT DNA analysis software.

The distribution of NPE cells to the phases of the cell cycle was analysed in bicuculline-treated and control cells. The cells were stained with PI to visualise DNA content/cell and run on a flow cytometer. The results were analysed by the ModFit LT DNA analysis software. The results showed that 22% of the control cells (1 µM GABA; Fig. 4A; n = 4) and 22% of the bicuculline-treated cells (Fig. 4B; n = 4) were in either S or G_2_/M phases.

### CDI p27^KIP1^ and p21^CIP^ expression in bicuculline-treated NPE cells

**Figure 5:**
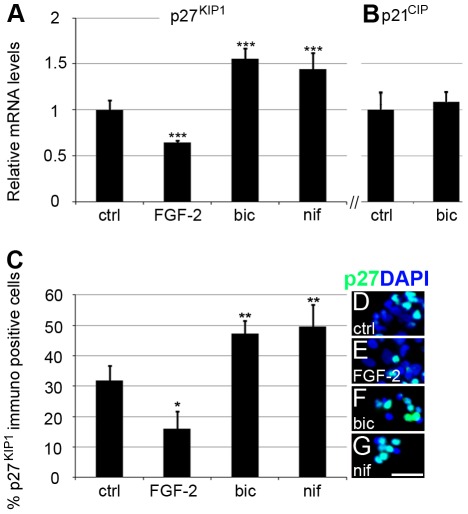
Expression of cyclin-dependent protein kinase inhibitors p27^KIP1^ and p21^CIP^ in cultured NPE cells. mRNA levels in cultured NPE cells were analysed by qRT-PCR and normalised to β-actin and TATA box binding protein. Relative expression of (A) p27^KIP1^ in control (1 µM GABA), FGF-2- (1.5 µg/ml), bicuculline- (20 µM bicuculline, 1 µM GABA), or nifedipine-treated (10 µM nifedipine, 1 µM GABA) cells. (B) Relative mRNA levels of p21^CIP^ in control (1 µM GABA) or bicuculline-treated cells (20 µM bicuculline, 1 µM GABA). Fixed dissociated cells were stained with the nuclear stain DAPI and an antibody against p27^KIP1^. (C) Percentage of p27^KIP1^ cells in the control, FGF-2-, bicuculline- and nifedipine-treated cells. Error bars ±SD, n = 4 independent cultures. Statistical test was one-way ANOVA, Tukey's multi-comparison post hoc test; p<0.05*, p<0.01**, p<0.001***. Representative images of p27^KIP1^ immunoreactivity among (D) control, (E) FGF-2, (F) bicuculline- or (G) nifedipine-treated cells. Scale bar in (G) is 25 µm and is also valid for (D)–(F). bic, bicuculline; ctrl, control; FGF-2, basic fibroblast growth factor; nif, nifedipine.

p27^KIP1^ and p21^CIP^ are endogenous cyclin-dependent kinase (CDK) inhibitors that regulate late G_1_ cyclin-CDK and S phase cyclin-CDK activity [36], [37]. Analysis of the expression of p27^KIP1^ and p21^CIP^ in control (1 µM GABA) and bicuculline-treated NPE cells showed a 1.6-fold increase of p27^KIP1^ mRNA in the bicuculline-treated cells compared to control cells (Fig. 5A). In contrast, no difference was seen for the p21^CIP^ mRNA levels (Fig. 5B). The p27^KIP1^ expression was also quantified by counting p27^KIP1^ immunocytochemistry-positive cells (4 independent cultures). 32±5% (935 of 2942 cells; n = 4) of control cells and 47±4% (1004 of 2121 cells; n = 4) of the bicuculline-treated cells were positive for p27^KIP1^ immunoreactivity (Fig. 5C, D and F).

### Nifedipine up-regulates p27^KIP1^expression and decreases NPE cell proliferation

It has previously been shown that the embryonic depolarising GABA system increases intracellular Ca^2+^ concentration through activation of the L-type voltage gated calcium channels (VGCC) [38]–[41]. Increased intracellular Ca^2+^ induces the expression of immediate early genes, including c-fos [42]. This in turn regulates the p27 ^KIP1^ expression, other mitotic regulators, and the cell cycle progression [43]. NPE cells were therefore treated with the specific L-type VGCC blocker nifedipine, which resulted in a 1.85-fold lower incorporation of [^3^H]-thymidine compared to control (1 µM GABA; n = 4) (Fig. 2B). The expression of p27 ^KIP1^ mRNA in control, nifedipine- and FGF-2-treated cells was then examined. The nifedipine-treated cells had 1.4-fold higher (n = 4) and FGF-2-treated cells had a 1.5-fold lower (n = 4) p27 ^KIP1^ mRNA levels than control cells (Fig. 5A). The p27^KIP1^ levels in the nifedipine-treated cells were comparable with those in the bicuculline-treated cells. The changes in p27^KIP1^ mRNA expression were confirmed by immunocytochemistry for p27^KIP1^ and cell-counting showing that 50±7% (2357 of 4745 cells; n = 4 cultures) of the nifedipine-treated cells, 32±5% of control and 16±6% (165 of 1023 cells) of FGF-2-treated cells were p27^KIP1^ positive (Fig. 5C–E, G).

### KCl counters the decreased proliferation caused by bicuculline

The nifedipine results support the idea that the depolarised NPE membrane potential is associated with cell cycle regulation. Control NPE cells (1 µM GABA) and bicuculline-treated cells were grown in KCl in order to depolarise the cells [44]. 100 mM KCl had a negative effect on the proliferation of both control and bicuculline-treated cells (data not shown). Increasing extracellular concentration of KCl from 4 to 20 mM did not have any effects on the proliferation of control cells but reduced the negative effect on the proliferation caused by bicuculline from 46% (bicuculline+1 µM GABA; n = 4) to 74% (20 mM KCl+bicuculline+1 µM GABA; n = 4) of the control cells (Fig. 2B). The results supported the hypothesis that treatment with bicuculline modulates the membrane potential, which has a negative regulatory effect on cell proliferation.

### Expression of γ-H2AX and effects of inhibitors of ATM/ATR and Chk1 on NPE cell proliferation

GABA_A_ receptor activation was recently shown to limit the proliferation of adult neural stem cells by recruiting the PI3K-related kinase pathway and histone H2AX phosphorylation (γ-H2AX) [45]. NPE cells were therefore treated with bicuculline and the number of γ-H2AX positive cells were analysed by immunocytochemistry. However, there was no difference in the expression of γ-H2AX between bicuculline-treated and control cells. Cells were also treated with inhibitors of ATM/ATR kinases (CGK 733) and checkpoint kinase 1 (Chk1; SB-218078). None of these inhibitors provided any consistent effects on the NPE cell proliferation. As a positive control neocarzinostatin was used. This is a radiomimetic agent known to trigger the γ-H2AX and ATM/ATR kinases and the response was robust: >50% of the cells were positive for γ-H2AX (data not shown).

## Discussion

During the early development of the nervous system, GABA_A_ receptor mediated signalling is involved in a variety of processes from cell proliferation and migration, via dendritic and axonal outgrowth, to synapse formation and plasticity [32]. The main focus of this work was the GABA_A_ receptor system and its effects on the proliferation of one of the sources of stem cell-like cells in the eye, the NPE cells of the ciliary body. The cells were studied because they can be prepared as a relatively homogenous cell sample in sufficient numbers to perform the different analyses in this study and because of that they are a potential source of cells for therapeutic purposes. The results from our study suggest that GABA maintains the proliferative potential for these cells. The GABA_A_ receptor expression with α1, α4, β2 and γ2 as the major subunits is consistent with extrasynaptic receptor assemblies and tonic properties [46]. 1 µM GABA maintained the proliferation of the cells *in vitro*. Increasing concentration of GABA or adding the GABA_A_ receptor agonist muscimol had no further stimulating effect on the tonic currents or the proliferation (Fig. 1 and Fig. 2). Antagonists of the GABA_A_ receptors decreased the proliferation (Fig. 2) without causing cell death or irreversible effects.

**Figure 6:**
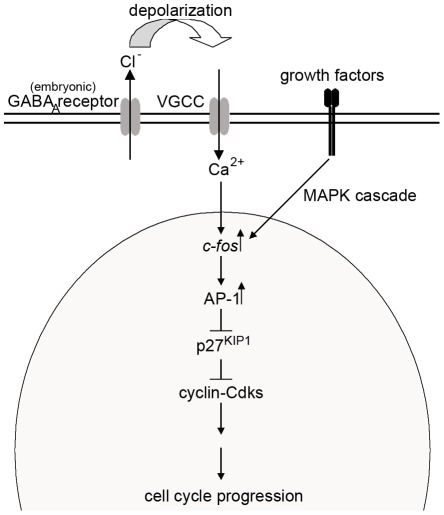
Schematic image illustrating the proposed mechanism how embryonic depolarising GABA_A_ receptors and L-type VGCCs regulate proliferation. Activation of GABA_A_ receptors generates a depolarised plasma membrane and activation of L-type voltage-gated Ca^2+^ channels (VGCCs). The intracellular Ca^2+^ concentration increases and leads to up-regulation of immediate early genes [42]. c-Fos and c-Jun form the Activator Protein-1 (AP-1) complex that represses the expression of cyclin-dependent protein kinase inhibitors (p27^KIP1^) and cyclin-cyclin dependent kinases (Cdks) are free to promote cell cycle progression. Growth factors work through the same pathway to stimulate the expression of immediate early genes.

The expression of KCC2, outward Cl^−^ transporter, was low and NKCC1, inward Cl^−^ transporter, was relatively high in the cells (Fig. 1), consistent with the cells having relatively high intracellular Cl^−^ concentration [47]–[49]. Inhibition of the GABA_A_ receptor Cl^−^ channels should thus prevent Cl^−^ efflux and prevent depolarisation of the membrane potential [32], [50]. The effects on proliferation by the GABA_A_ receptor antagonists could be counteracted by addition of extracellular KCl (Fig. 2), a treatment that depolarises the plasma membrane [44]. Inhibition of the L-type VGCCs also reduced the proliferation of the NPE cells in a similar fashion to the GABA_A_ receptor inhibitors. These results are consistent with that the membrane potential of the NPE cells is important for maintaining cell proliferation, and when the resting potential is maintained the cells do not proliferate [17]. The increased expression of the CDI p27^KIP1^ after inhibition of either the GABA_A_ receptors or the L-type VGCCs suggests a link between GABA_A_ receptors, membrane depolarisation, and VGCCs in the regulation of the cell cycle (Fig. 6). It is well established that Ca^2+^/calmodulin stimulates the expression of genes involved in the cell cycle progression by Ca^2+^/calmodulin-dependent protein kinases and protein phosphatase calcineurin [51]. Bicuculline seems to have slowed down the cell cycle rather than arresting it in a specific phase (Fig. 4). This is consistent with that the sustained Ca^2+^/calmodulin act at several phases of the cell cycle, including the initiation of the S phase, and both initiation and completion of the M phase [51]–[53]. The NPE cells responded to FGF-2 with an increased proliferation accompanied with a decreased CDI p27^KIP1^ expression. This is in accordance with the well-known mitogenic actions by growth factors [42]. VGCCs also induce immediate early genes [42] that down-regulates the expression of p27^KIP1^ [43]. The expression of p27^KIP1^ was higher in nifedipine-treated NPE cells compared to control cells (Fig. 5). Thus inhibition of the GABA_A_ receptors and/or VGCCs stimulated the expression of p27^KIP1^, whereas stimulation with FGF-2 inhibited the p27^KIP1^ expression (Fig. 5). Based on these results we propose that extrasynaptic GABA_A_ receptors on NPE cells regulate the membrane potential, and modulates the open probability of VGCCs resulting in regulation of intracellular Ca^2+^ concentration; and, thereby, keeping the NPE cells in a permissive state for proliferation (Fig. 6).

In addition to the regulatory pathway with Ca^2+^ and the MAPkinase cascade via L-type VGCCs [21], [54], GABA_A_ receptors have been shown to recruit the PI3K-related ATM/ATR pathway for epigenetic regulation of proliferation of embryonic stem cells and adult neural progenitors [19], [45]. The present work supports the recruitment of L-type VGCCs and Ca^2+^ but not of the ATM/ATR kinases or phosphorylation of H2AX. The expression of γ-H2AX or p21^CIP^ was not increased and inhibitors of ATM/ATR kinases or check-point kinase-1 did not counteract the reduction of the proliferation of cells treated with bicuculline.

*In vivo*, NPE cells are contact inhibited and mitotically quiescent, which have been shown for retinal pigment epithelium and most other epithelia [55], [56]. Proliferation can be triggered by exogenous growth factors [4] or as this study showed, by dissociation and culturing in medium without growth factors. The cells of the neighbouring CMZ, also known as the *ora serrata* or the circumferential germinal zone [10], proliferate throughout late embryonic development and during early post-hatch (post-natal) period [3], [35]. As the retina grows rapidly in size, we speculate that NPE cells from the *pars plana*, which is the part of the ciliary body that is located closest to the CMZ [4], may contribute to the proliferating cells in the CMZ. The CMZ is heterogeneous with retinal progenitors in different stages of differentiation with the least developed cells next to the NPE [15]. It was recently suggested based on c-myc expression in the frog retina that cells in the far peripheral CMZ, next to the NPE, are candidates for a niche-dependent population of retinal stem cells that give rise to rapidly dividing progenitors of limited proliferative potential [57]. The results in this study showed that GABA_A_ receptor inhibition robustly decreased the proliferation of the E12 prospective CMZ cells (Fig. 3). We therefore suggest that low concentrations (submicromolar) of GABA contribute to maintaining the proliferation of not only NPE cells but also of progenitor cells in the prospective E12 CMZ. Low ambient concentrations of GABA may come from endogenous synthesis in NPE or ciliary body cells, as suggested by the expression of GAD67 (Fig. 1), or from cells in the mature retina directly or via the vitreous. Vitreous fluid has been shown to contain low levels of GABA [58], [59]. The glucagon expressing “bullwhip” amacrine cells [60], [61] have been proposed to regulate the proliferation of progenitor cells within the CMZ and NPE [62]. The “bullwhip” cells are Ap2α+ and GABA-negative [61] indicating that they do not contribute with GABA to directly regulate the GABA_A_ receptor mediated effects on proliferation as shown in this study. The GABA-ergic cells in the prospective CMZ were Ap2α-negative but Pax6+ and Isl1+ (Fig. 3H–J), suggesting that the cells either were GABA producing ganglion cells or Ap2α-negative displaced amacrine cells [63].

In more central parts of the E12 retina there was no robust reduction of the proliferation by treatment with bicuculline. This indicates that late retinal progenitor cells respond differently from progenitors in the developing NPE and CMZ. Moreover, there were no significant effects of bicuculline on the early progenitors in E3.5 or E5 retinas (supplementary Fig. S1). However, an effect was seen on progenitors in central parts of the E8 retina (Fig. 3) suggesting that the response to GABA by retinal progenitors is stage dependent. We speculate that the unresponsiveness by early retinal progenitor cells may reflect that the GABA_A_ receptor expression is low before E8 (st35) [26]. Furthermore, at E8 the depolarising action of GABA reaches a peak and after E12 GABA assumes its classical inhibitory action. At E14 GABA does not longer induce calcium influx [64]. In addition to this, GABA synthesis appear to be low before E6 [65]. This may explain the different effects of GABA_A_ receptor inhibition on the proliferation of progenitor cells of different ages. Similar results with both increased and decreased cell numbers after GABA receptor antagonist treatment have been obtained in studies of the neocortical ventricular and subventricular zones [66]. The variable responses of stem and progenitor cells to GABA are likely to reflect several properties of the target cells. There are many factors that may contribute to the variable responses, for example the GABA_A_ receptor expression, receptor subunit composition, factors regulating intracellular Cl^−^ and/or Ca^2+^ concentrations, and the Ca^2+^ sensing pathways that regulate gene expression and the cell cycle.

This study concludes that chicken NPE cells and certain retinal progenitors have functional GABA_A_ receptors that contribute to the regulation of the cell proliferation. We propose that the embryonic GABA_A_ receptors contribute to maintain the proliferation by regulating the plasma membrane potential. This has been shown to be important in many developmental processes, for example the patterning of the visual field [67].

## Supporting Information

Figure S1 **Effects of bicuculline on E3.5 and E5 retinal progenitor cell proliferation.** Fluorescence micrographs of EdU labelled cells in (A, D, F) control and (B, E and G) bicuculline-treated E3.5 (A)–(B) or E5 (D)–(G) retinal explants cultured for 4 hours. Images in (a) to (f) show the boxed regions in (A) and (B) in higher magnification. (C) Bar graph shows the cell density of EdU positive cells in central and peripheral retina in control (black bars) or bicuculline-treated (grey bars) E3.5 explants. Error bars ± S.D. n = 3 explant cultures, average of 4 sections per explant, Mann-Whitney test; n.s. p = 0.34 (central) and p = 0.17 (peripheral). (D) and (E) show the central E5 retina whereas (F) and (G) show the peripheral. (H) Bar graph shows the cell density of EdU positive cells in central and peripheral retina in control (black bars) and bicuculline-treated (grey bars) E5 explants. Error bars ± S.D. n = 3 explant cultures, average of 4 sections per explant, Mann-Whitney test; n.s. p = 0.35 (central) and p = 0.1 (peripheral). bic, bicuculline; n.s., not significant. Scale bar in (B) is 200 µm and is also valid for (A). Scale bar in (G) is 100 µm and is also valid for (D)–(F).
(TIF)
