# Supplementary figures and images for: GABA Maintains the Proliferation of Progenitors in the Developing Chick Ciliary Marginal Zone and Non-Pigmented Ciliary Epithelium
Source: PLoS One. 2012 May 9;7(5):e36874. doi: 10.1371/journal.pone.0036874 (PMC3348890; doi:10.1371/journal.pone.0036874)

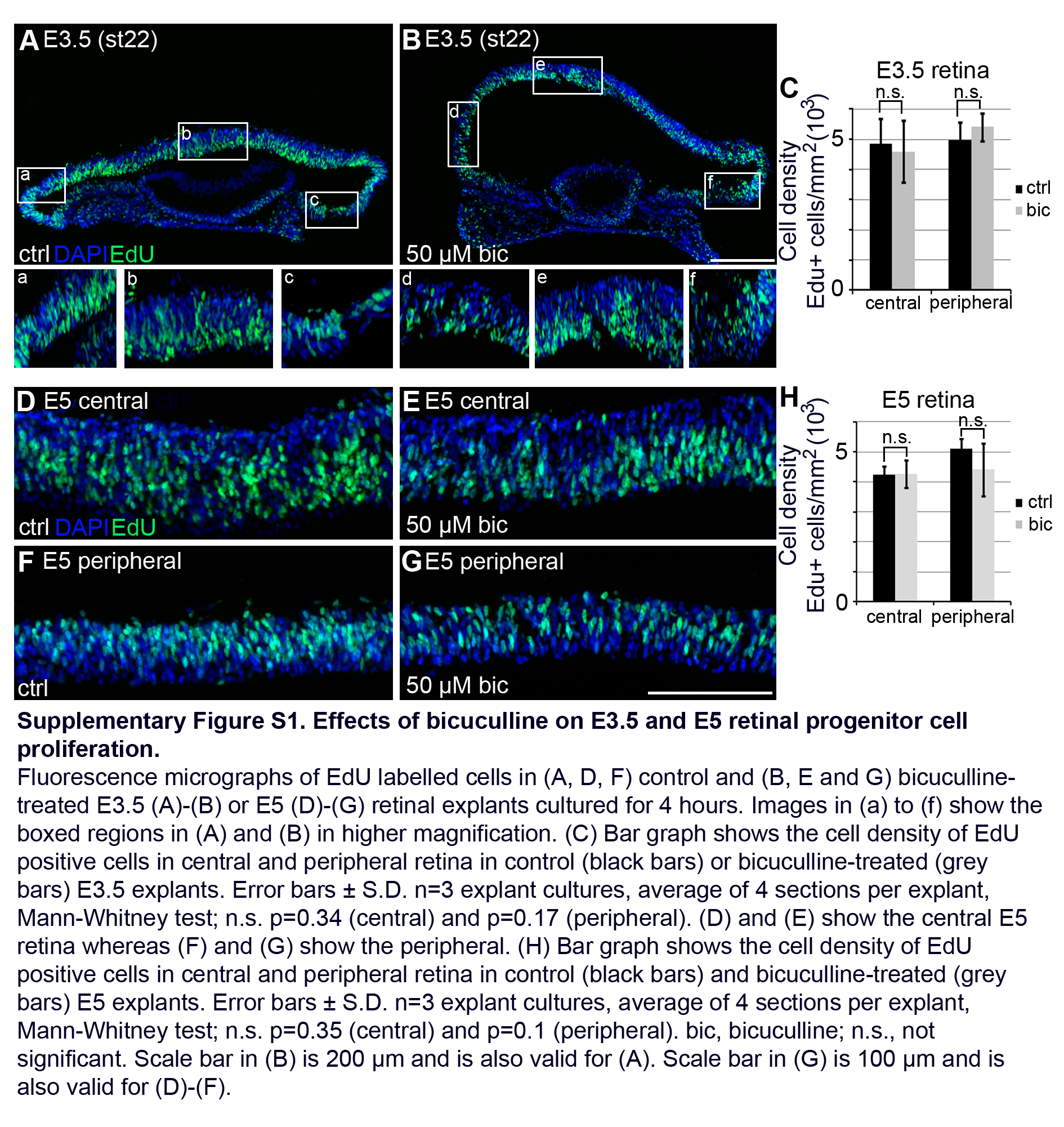

Supplement: Figure S1 — Effects of bicuculline on E3.5 and E5 retinal progenitor cell proliferation. Fluorescence micrographs of EdU labelled cells in (A, D, F) control and (B, E and G) bicuculline-treated E3.5 (A)–(B) or E5 (D)–(G) retinal explants cultured for 4 hours. Images in (a) to (f) show the boxed regions in (A) and (B) in higher magnification. (C) Bar graph shows the cell density of EdU positive cells in central and peripheral retina in control (black bars) or bicuculline-treated (grey bars) E3.5 explants. Error bars ± S.D. n = 3 explant cultures, average of 4 sections per explant, Mann-Whitney test; n.s. p = 0.34 (central) and p = 0.17 (peripheral). (D) and (E) show the central E5 retina whereas (F) and (G) show the peripheral. (H) Bar graph shows the cell density of EdU positive cells in central and peripheral retina in control (black bars) and bicuculline-treated (grey bars) E5 explants. Error bars ± S.D. n = 3 explant cultures, average of 4 sections per explant, Mann-Whitney test; n.s. p = 0.35 (central) and p = 0.1 (peripheral). bic, bicuculline; n.s., not significant. Scale bar in (B) is 200 µm and is also valid for (A). Scale bar in (G) is 100 µm and is also valid for (D)–(F). (TIF) [file pone.0036874.s001.tif]
